# Bone density may affect primary stability of anterior cruciate ligament reconstruction when organic core bone plug fixation technique used

**DOI:** 10.1186/s40634-021-00441-z

**Published:** 2022-01-06

**Authors:** Pouya Dehestani, Farzam Farahmand, Amirhossein Borjali, Kaveh Bashti, Mahmoud Chizari

**Affiliations:** 1 Department of Mechanical Engineering, Sharif University of Technology, Tehran, Iran; 2 Tehran University of Medical Sciences, Tehran, Iran; 3 Department of Orthopedics, Division of Knee Surgery, Shariati Hospital, Tehran University of Medical Sciences, Tehran, Iran; 4 Present Address: School of Physics, Engineering and Computer Sciences, University of Hertfordshire, College Ln, Hatfield, AL10 9AB UK

**Keywords:** Core bone plug fixation, ACL reconstruction, Bone density, Implant-less, Tensile test

## Abstract

**Purpose:**

Core Bone Plug Fixation (CBPF) technique is an implant-less methodology for ACL reconstruction. This study investigates the effect of bone density on CBPF stability to identify the bone quality that is likely to benefit from this technique.

**Methods:**

Artificial blocks with 160 (Group 1), 240 (Group 2), and 320 (Group 3) kg/m^3^ densities were used to simulate human bone with diverse qualities. These groups are representative of the elderly, middle age and young people, respectively. A tunnel was made in each test sample using a cannulated drill bit which enabled harvesting the core bone plug intact. Fresh animal tendon grafts were prepared and passed through the tunnel, so the core bone was pushed in to secure the tendon. The fixation stability was tested by applying a cyclic load following by a pullout load until the failure occurred. The selected group was compared with interference screw fixation technique as a gold standard method in ACL reconstruction.

**Results:**

The Group 2 stiffness and yield strength were significantly larger than Group 1. The graft slippage of Group 1 was significantly less than Group 3. The ultimate strengths were 310 N and 363 N, in Groups 2 and 3, significantly larger than that of Group 1. The ultimate strength in fixation by interference screw was 693.18 N, significantly larger than the bone plug method.

**Conclusions:**

The stability of CBPF was greatly affected by bone density. This technique is more suitable for young and middle-aged people. With further improvements, the CBPF might be an alternative ACL reconstruction technique for patients with good bone quality.

**Clinical relevance:**

The CBPF technique offers an implant-less organic ACL reconstruction technique with numerous advantages and likely would speed up the healing process by using the patient’s own bones and tissues rather than any non-biologic fixations.

## Introduction

ACL rupture is one of the most common knee injuries worldwide [1]. Surgical treatment, i.e., ACL reconstruction, involves the replacement of the deficient tissue with a natural or synthetic graft. However, fixation of the graft is problematic, especially at the tibial side with a lower density [1]. A wide variety of graft fixation methods and devices have been developed in the past, each having its own advantage/disadvantage. Currently, interference screw fixation, by either an absorbable or a metallic implant, is considered the most popular technique for securing the graft [2, 3]. However, in addition to the high costs of the implant, there are several side effects associated with this technique that can affect the long term performance of the reconstruction, e.g., implant corrosion and body reactions [4–7], tunnel enlargement [8], may require a second operation for removal [9], inflammatory action [10, 11], blocking the blood transfer to the graft that prevents tendon feeding [11], intraarticular migration of the implant [12, 13], producing acidic, the low absorption rate in some cases [9, 14], and finally making defects in the CT and MRI photography [15]. The main cause of all such side effects is the employment of a non-biologic fixation device, i.e., metal interference screw, for graft fixation.

**Fig. 1:**
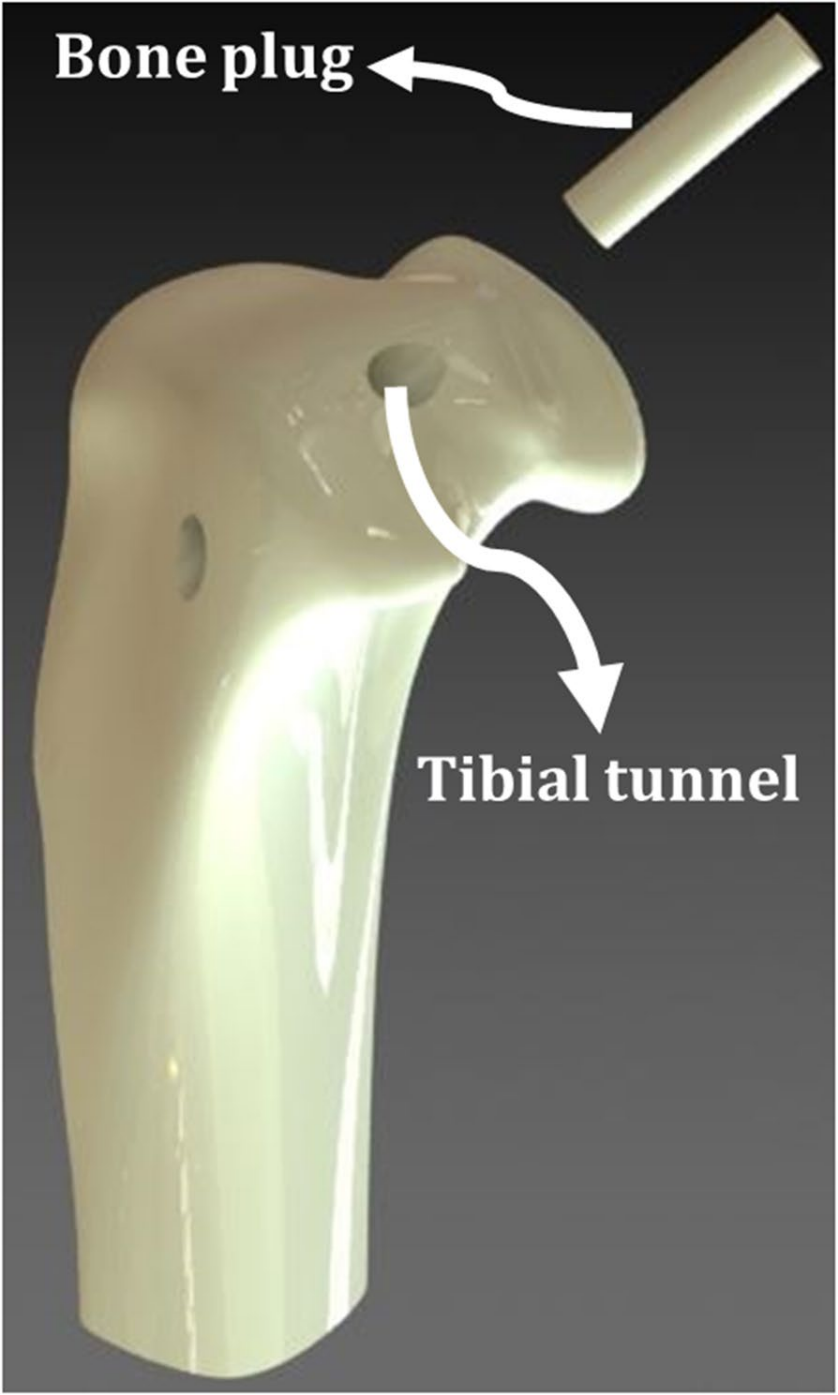
ACL reconstruction using Core Bone Plug Fixation (CBPF) technique. The bone plug is cut and harvested during bone tunnelling

The Core Bone Plug Fixation (CBPF) technique (Aka Bone and Site Hold Tendon Inside (BASHTI) technique) is a newly proposed methodology for ACL reconstruction. Hence, the method is implant-less, which does not use any external object for graft fixation [16]. Furthermore, the technique is organic and using the patient’s own bone and soft tissue. By utilizing a cannulated drill bit, tunnels are made at the graft fixation locations in the tibia or femur, with the core bone cut at the same time and extracted intact from the tunnels (Fig. 1). The resulted core bone plug is then used to be placed between the tendon graft strands and then inserted into the tunnels using a hand hammer.

As such, the graft fixation is performed using the patient’s own bone, with no need for a non-biologic fixation device. Although not clinically examined yet, the CBPF is expected to provide improved graft-bone integration compared with the interference screw fixation. This expectation is based on the analogy of this technique with the ACL reconstructive surgery using bone-patellar tendon-bone autograft, for which very successful long-term clinical results have been reported [17, 18].

A sufficiently high primary stability is essential for the short- and long-term success of an ACL reconstruction [19]. Excessive motions at the graft-bone interface, caused by a poor primary fixation, may lead to early graft pull-out or postpone or even prevent the secondary fixation achieved by graft-bone integration. A previous biomechanical study on animal samples has shown that the primary stability of CBPF is reasonable and comparable with those absorbable interference screws [16]. However, a significant deviation was observed in the results, presumably due to the uncontrolled bone samples that had diverse densities. That is why the study suggested performing a biomechanical examination with a controlled bone density.

The effect of geometry parameters such as tunnel size, core bone plug diameter, and tendon diameter on the strength of CBPF has been investigated using finite element models and experimental tests [20–24]. The results showed that the mode of the tendon failure and fixation strength were highly influenced by the tendon compression and core bone diameter. Furthermore, an experimental study concluded that using a sheathed core bone plug makes the insertion process more feasible and improves the CBPF or BASHTI technique more reliable [25]. The CBPF technique has also been used to apply the biceps tenodesis or long head biceps repair, and the results were comparable with other conventional biceps tenodesis methods [26, 27].

This study aims to investigate the effect of bone density on the primary stability of the CBPF in detail. Pull-out tests are performed on the constructed graft-bone samples, modelled by fresh bovine digital extensor tendon substitute for the human hamstring tendon, and artificial sanitary polyurethane Sawbones blocks (Sawbones Europe AB, Sweden) substitute for the human tibial bone. Different densities of Sawbones blocks are used to evaluate the stiffness, yield strength, and ultimate fixation strength. In addition, an interference screw technique was implemented to compare the results of this method with the CBPF method. Finally, knowing about relative motion between components at the fixation site is an interest of the study.

## Materials and methods

### Tendon graft preparation

**Fig. 2:**
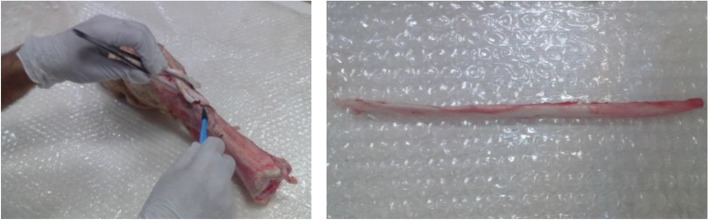
Tendon harvesting procedure from a bovine hoof and a harvested tendon strand

**Fig. 3:**
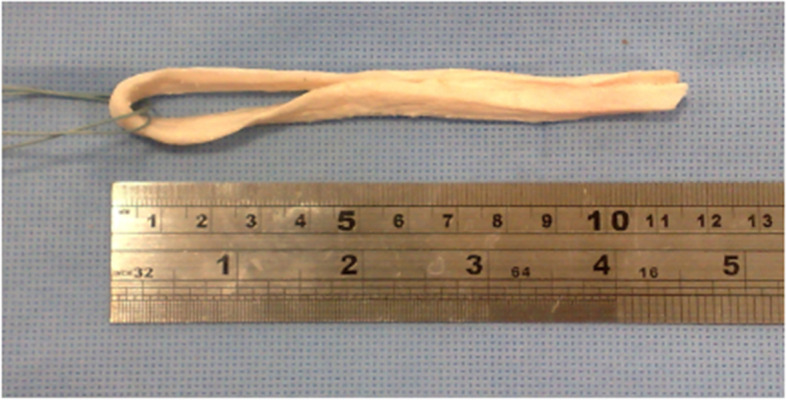
A typical looped bovine tendon sample is used as a soft tissue graft for experiments

Forty fresh bovine digital extensor tendons were used as the soft tissue tendon grafts for the experimental purpose of the study. The tendons were harvested from the same race and same body conditions bovines. The bovines aged 9-month when those sent to an approved slaughtering house. The bovine hooves were cleaned, and the digital tendons were harvested in a biomechanical laboratory. The harvested tendon was then cut to the approximate length of 220 mm. Geometric aspects of prepared tendon samples have been summarized in Table 1. The process of tendon strand harvest from a bovine hoof has been illustrated in Fig. 2. The harvested tendons were then stored at − 22 °C for not more than 1 month before the experiments. At the time of the experiment, the samples were thawed at a room temperature of 26 °C and humidity of %28 and cleared from the surrounding tissues. The grafts were prepared by trimming the tendons to a specific diameter using a gage and making those to a loop fashion with a length considering the length of the tunnel and the 30 mm gage length needed to model an ACL reconstruction method. A typical looped graft has been shown in Fig. 3. The tendons were kept moist throughout the experiments using pure water spray.

**Table 1:** Geometric aspects of prepared tendon samples for experiments

Tendon strand size	220 mm (± 10) mm length;
**Looped tendon graft size**	7.5 mm diameter; 110 (± 1.5) mm looped length

### Core bones preparation

Sawbones blocks with different densities were used in analogy to the human bone with diverse qualities. The similarity of the mechanical properties of Sawbones blocks of different densities with those of the cadaver tibias at different ages has been established in previous studies [28]. Wilks et al. [29] showed that the proximal tibia bone density of middle age in both male and female genders (about 45 years old) is approximately 240 kg/m3, which is in line with what we have used as a bone density of the subject patients. This can be considered for all patients who are not suffering from any bone disease. Therefore, three groups of low density (160 kg/m^3^), middle density (240 kg/m^3^), and high density (320 kg/m^3^) Sawbones blocks were used in association with the bone quality of old, middle age and young human subjects, respectively. The blocks were used in the original size of 180x130x40 mm with no cut. In addition, looped tendon grafts with a 7.5 mm diameter were chosen for experiments. Ten tunnels were made at each block using a custom-made cannulated drill bit with 9 mm internal and 10 mm external diameters. After the tunnelling and extracting the core bone from the cannulated drill bit, the core bone head was chamfered to a conical shape using 80-grit sandpaper. This will help the core to be inserted into the tunnel more easily. The overall integrity of the core bone plugs, formed as a result of tunnelling, was investigated by visual observation. Before the experiment, the geometry of all components was checked using a micrometre. The mean diameter and standard deviations of the tunnels were 10.3 (± 0.07) mm, and the core bone was 8.8 (± 0.018) mm. Detailed information on the core bone and the tunnel has been summarized in Table 2.

**Table 2:** Geometric aspects of prepared components for experiments

	Group 1	Group 2	Group 3
**Sawbones block density**	160 kg/m^3^	240 kg/m^3^	320 kg/m^3^
**Sawbones block size**	180x130x40 mm; same for all groups		
**Core bone size**	8.8 (± 0.018) mm diameter; 40 (± 0.08) mm length		
**Tunnel size**	10.3 (± 0.07) mm diameter; 40 (± 0.02) mm depth		

### Screw preparation

**Fig. 4:**
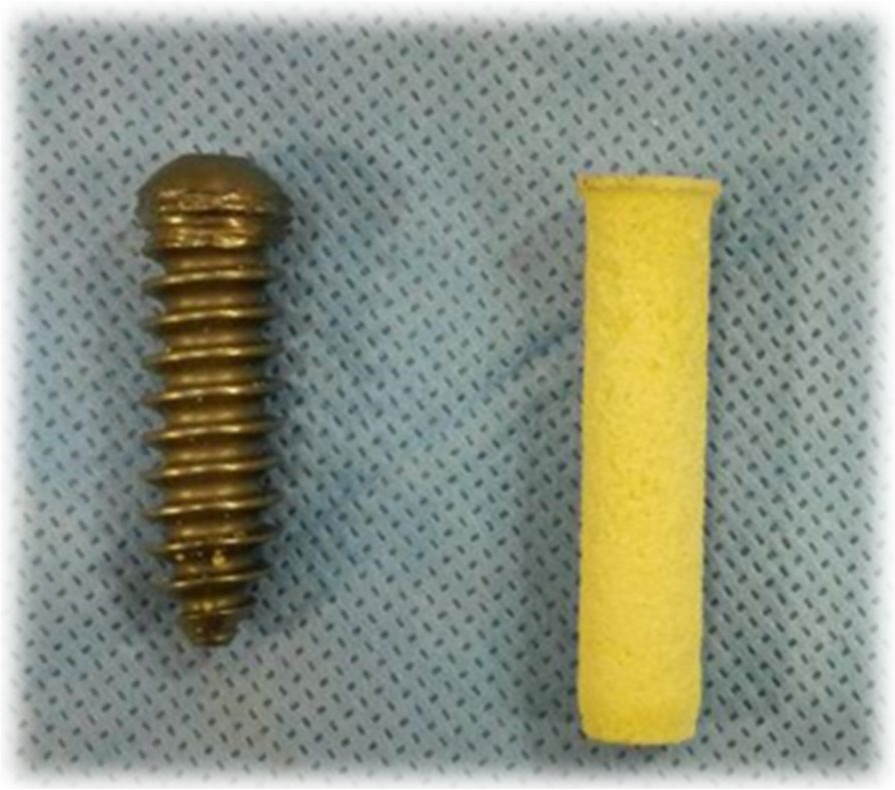
RCI metal screw which is used for fixation in the tests and their size in comparison with a bone plug

The RCI screw from Smith & Nephew Company was used for fixation (Fig. 4). The diameter of the screw was 8 mm, and the overall length of it was 35.2 mm, 30 mm of which was threaded. Also, the tunnel size was considered 10 mm, which is similar to the CBPF technique.

### Insertion process

After the preparation of the components, the final specimens were constructed by inserting the tendon grafts into the blocks. The sample construction was performed as follows. Initially, the tendon strand was looped on a guide suture, as shown in Fig. 3. The looped tendon was then passed through the tunnel with the aid of the guide suture. The length of the looped portion of the tendon (gage length) was kept at 30 mm for all specimens.

**Fig. 5:**
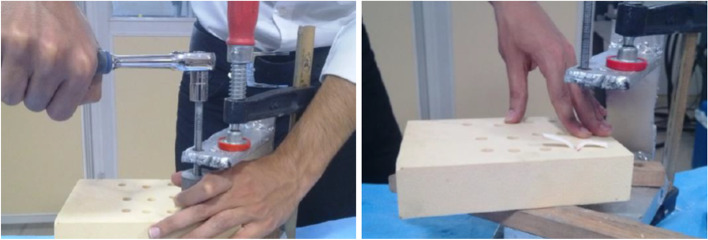
Insertion process of core bone into the tunnel; a guide cylinder supported the core bone against buckling or possible fracture

**Fig. 6:**
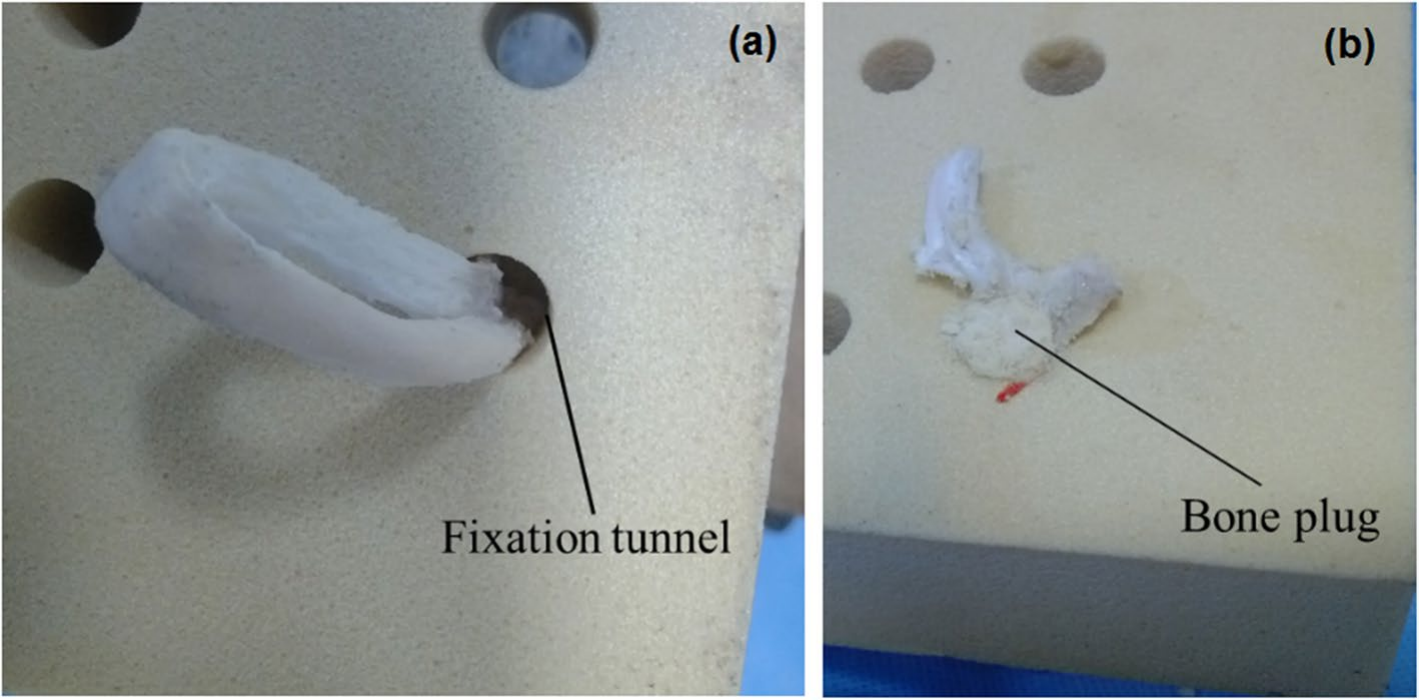
Top view **a** and bottom view **b** of the constructed sample using the CBPF technique

Next, in the tests with the CBPF technique, the chamfered head of the core bone was placed between the tendon strands at the tunnel entrance. In the final step, the core bone was inserted into the tunnel using an in-house made screw-driven rig. The core bone insertion process was performed very slowly for at least three quarters (30 mm) of its length. A guide cylinder held the core bone in-line with the tunnel and protected it against buckling or possible fracture. The rig was designed to force the core bone into the tunnel using a screw-driven system, as shown in Fig. 5. The top and rear view of the final sample has been demonstrated in Fig. 6.

**Fig. 7:**
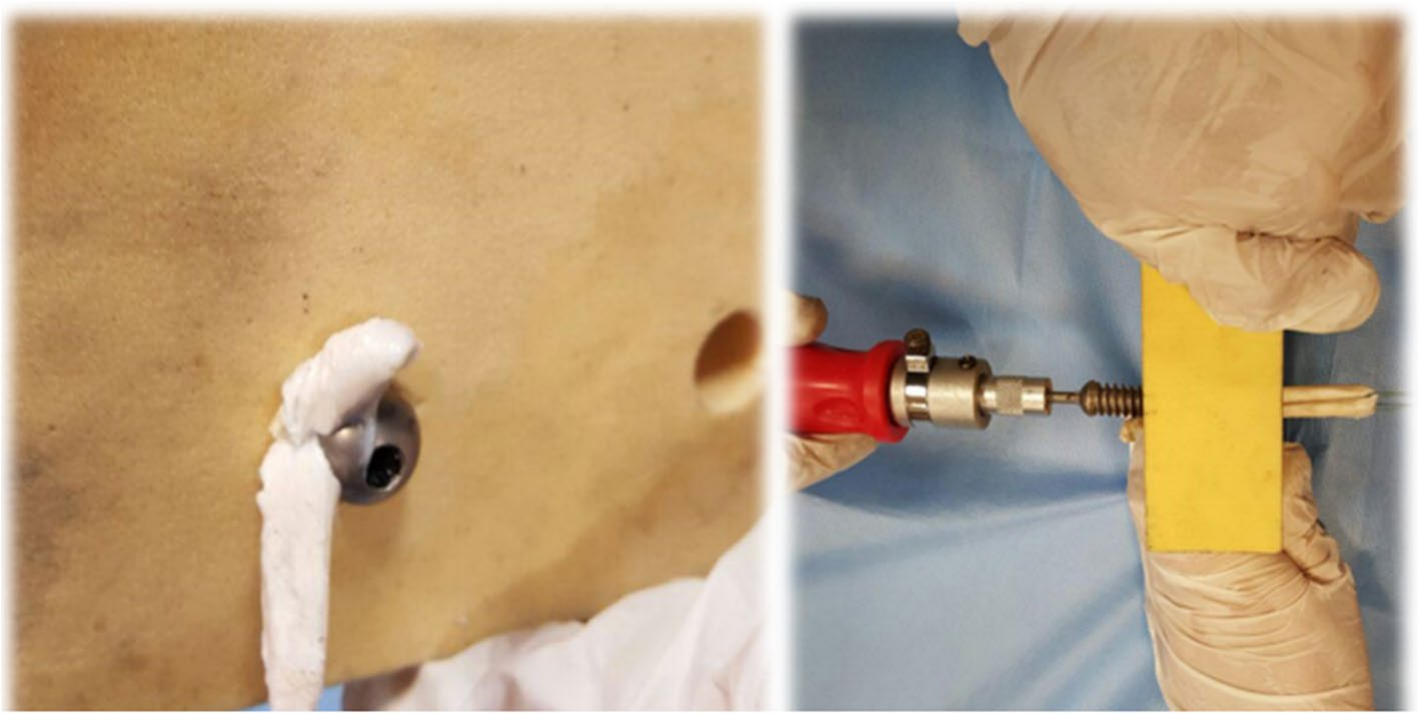
Placing the screw in the fixation tunnel and the sample of fixed graft in the tunnel by interference screw using Sawbones blocks with a density of 320 kg/m^3^

In conducting the experiments on the interference screw technique, we have attempted to conduct all the steps similar to those of the samples tested on the CBPF technique. But to place the screw inside the fixation tunnel, the tip of the screw is placed in the centre of the final part of the connective tissue, and then simultaneously with stretching the connective tissue from the other side, the screw was placed by the special screwdriver (Fig. 7). In order to do statistical analysis, the results of the mechanical properties of fixing the screw were similarly compared to the results of the third group of CBPF technique.

### Experimental setup

**Fig. 8:**
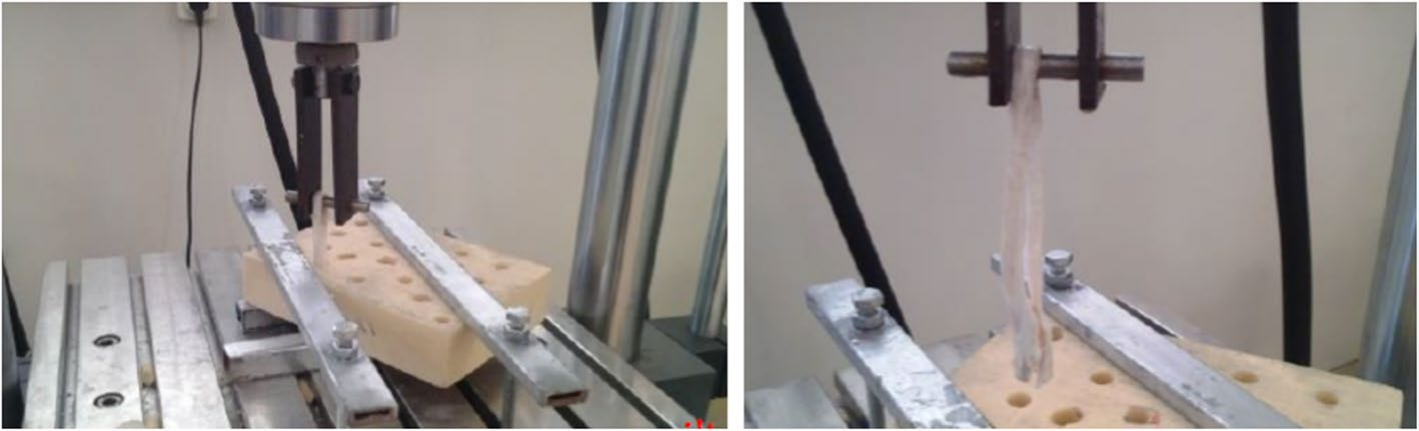
Mechanical testing of a sample mounted into a tensile testing machine

A tensile pull-out test was performed using a Zwick/Roell (Amsler HCT 25–400) testing machine to examine fixation strength. The specimen was mounted into the testing machine using a custom-made clamp, as shown in Fig. 8. The Sawbones block was located on the test stand, where the tunnel can be in line with the crosshead’s hanger. In the next step, the looped portion of the tendon was secured on the crosshead’s hanger. To make sure that the tendon fibres are tightened and bear similar loads, a preload of 5 N was applied to the specimen [28]. In continue, a sinusoidal preload of 5 to 20 N was applied for ten cycles with a frequency of 1.0 HZ for preconditioning. The tendon has a history-dependent behaviour [1]. The preconditioning process will justify the viscoelastic effect of the tendon graft. Immediately after the preconditioning, the specimen was subjected to the main test loading. At first, a cyclic sinusoidal load of 20 to 100 N with a frequency of 1.0 HZ for 100 cycles was applied to the specimen [1]. Then a single-cyclic failure load, with an incremental rate of 20 mm/s, was applied and continued until the fixation failed due to either the tendon graft rupture or the graft slips out of the tunnel. The mode of failure during the testing process was closely monitored. Figure 8 shows the pull-out testing process and the failed sample. At the end of each experiment, the overall integrity of the core bone was again investigated by visual observation.

## Results

### Cyclic test

**Fig. 9:**
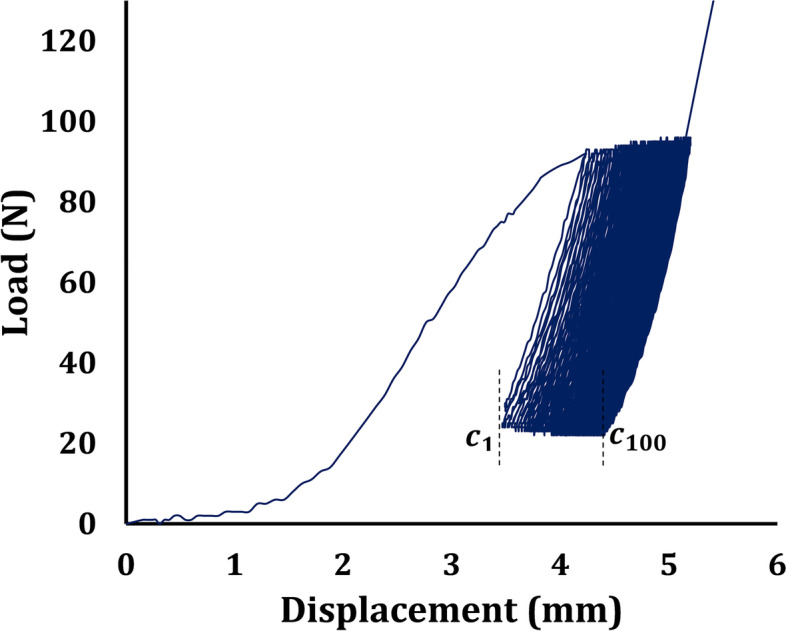
Force-displacement curve of the cyclic sinusoidal loading of a typical specimen. The graft slippage was determined as the displacement between c1-c100

The load-displacement data of the multicycle test is recorded for all tested samples. A typical force-displacement result obtained from the cyclic test is plotted in Fig. 9. The relative motion of the components at the fixation site or, in short, “graft slippage” was expected to be analysed. According to the previous study [30], fixation rigidity (graft slippage) equals the displacement difference between the first and hundredth oscillations in a cyclical loading, as shown in the following Fig. 9. If the rigidity of the structure exceeded 10 mm, then it could be said that the structure had lost its functionality, or the fixation failed [30]. It should be pointed here that the 10 mm threshold is considered as displacement of the structure. It means the definition is not considering the natural relaxation of the tendon and only focus on fixation itself [1].

The area under the curve indicates lost energy due to the internal friction of the material. It is noted that in cyclic tests the hysteresis loops gradually become slimmer, and after a specific number of loops, the curve stops moving forward. The lost energy reaches its minimum, and it shows that we have reached a stable condition and there would be no more creep in samples [31].

The means and standard deviations of the graft slippages of all test groups were obtained based on the data recorded during the cyclic tests. The outcome has been summarized for all four groups in Table 3. The results confirm that the samples with higher Sawbones density have smaller slippage at the fixation site.

**Table 3:** The means, standard deviations, and range of graft slippage results for all test groups

	Group 1 (160 kg/m^**3**^ density)	Group 2 (240 kg/m^**3**^ density)	Group 3 (320 kg/m^**3**^ density)	RCI screw 7–8 (320 kg/m^**3**^ density)
**Graft Slippage (mm)**	3.93 ± 3.46	3.49 ± 4.66	2.02 ± 1.70	1.24 ± 0.41

### Single-cyclic load to failure test

**Fig. 10:**
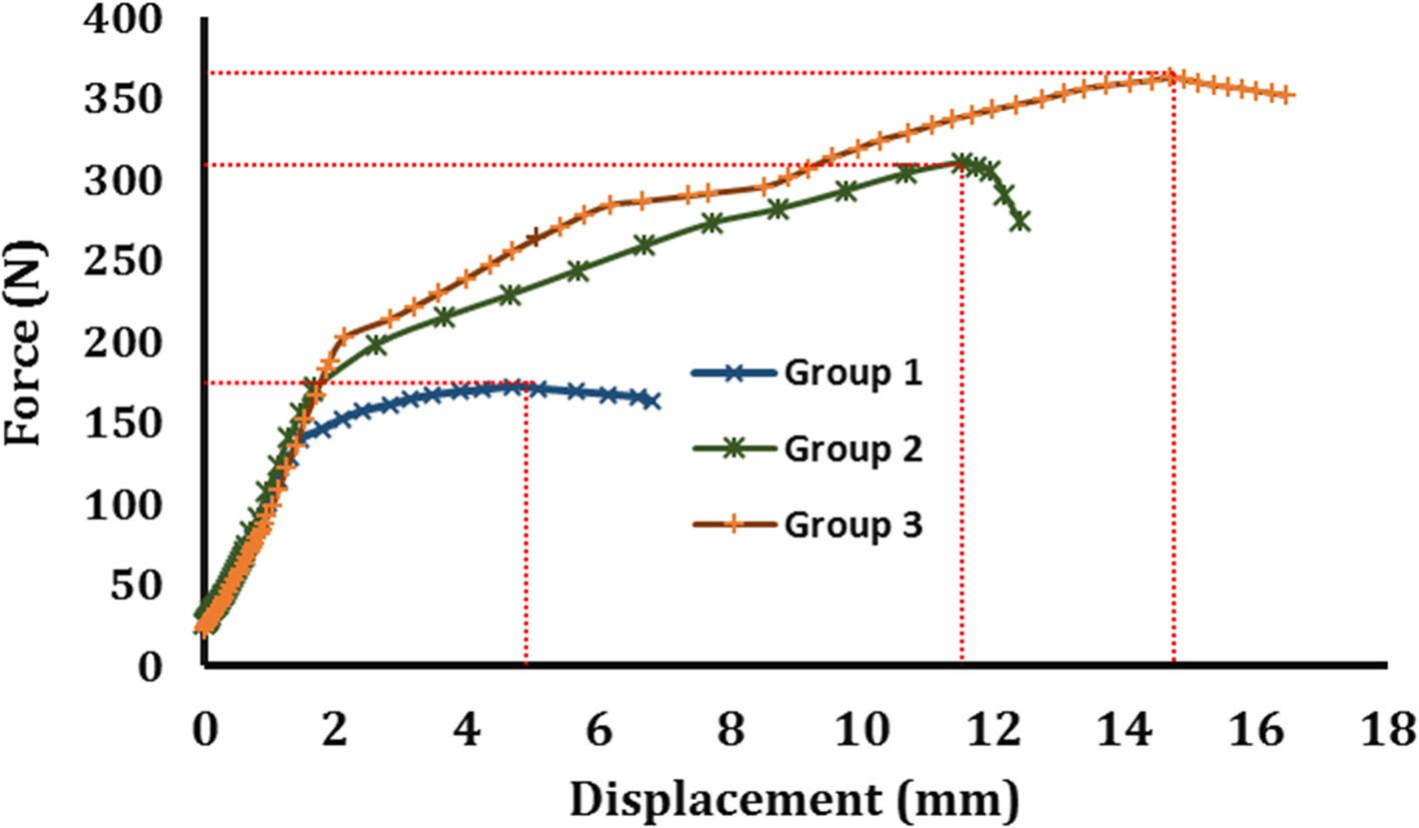
The averaged load-displacement curves of the three test groups during the failure loading tests. The ultimate strengths are shown in the figure

The average result of force-displacement of the three test groups of core bone fixation during the single cycle load to failure test has been illustrated in Fig. 10. Stiffness yield and the ultimate strength of the fixation were evaluated based on the experimental data for all test samples and summarized in Table 4. The yield strength was determined from the load-displacement curve as the endpoint of the elastic region where the slope started to decrease. The stiffness of fixation was defined as the slope of the curve in the elastic region. Finally, the ultimate strength of fixation was determined from the load-displacement curve as the maximum load that the sample can resist before failure. The results of the study were analysed in terms of means, standard deviations, and ranges. The student’s t-Test statistical analysis was performed to compare the results of the three test CBPF test groups and the interference screw fixation method. The difference was statistically significant if the probability measure was less the 0.05 (*p* < 0.05).

**Table 4:** The means, standard deviations, and ranges of primary stability characteristics of all test groups

	Group 1 (160 kg/m^**3**^ density)	Group 2 (240 kg/m^**3**^ density)	Group 3 (320 kg/m^**3**^ density)	RCI screw 7–8 (320 kg/m^**3**^ density)
**Stiffness(N/mm)**	76.7 ± 8.72^a^ (range: 61.6–92.4)	86.6 ± 8.56 (range: 67.4–102.7)	93.9 ± 26.13^c^ (range: 51.8–123.8)	158.9 ± 57.38 (range: 113.5–285.4)
**Yield Strength(N)**	139.7 ± 11.69^a^ (range: 120–162)	171.4 ± 28.92 (range: 140–231)	188.7 ± 82.37^c^ (range: 108–350)	416.02 ± 118.84 (range: 275–616)
**Ultimate Strength(N)**	172.2 ± 14.22^ab^ (range: 144–193)	310 ± 77.58 (range: 205–394)	363 ± 145.83^c^ (range: 205–702)	693.18 ± 143.95 (range: 532–942)

^a^Significantly different from group 2

^b^Significantly different from group 3

^c^Significantly different from screw fixation

Yield force is considered as the endpoint of the linear region at the displacement-force curve. The slope of the linear region, which was related to elastic deformation, was also considered as the value of fixation stiffness. However, the displacement at the yield point up to the ultimate force should be less than the threshold that was defined for failure. This displacement could be considered as fixation displacement [30, 32]. Therefore, if this displacement exceeds the value, the constructed graft might lose its function. This displacement may result in an excessive laxity in the body.

The means of stiffness increased with the increase of the density, up to 13% and 22% for groups 2 and 3, respectively, with reference to group 1. Although there was a deviation among the recorded data in each group, however, the stiffness deviation was considered significant (*p* < 0.05) only for groups 1 and 2, as indicated in Table 4. The stiffness results were mostly diverse for group 3, ranging from 51.8–123.8 N/mm among tested specimens. Similarly, the yield strength of fixation increased by increasing the density of Sawbones. The result among groups 1 and 2 was statistically significant (*p* < 0.05), as indicated in Table 4. The most considerable effect of the change of the density was associated with the ultimate strength of fixation. By changing the Sawbones density, the ultimate strength increased significantly. The change reference to group 1 was up to 80% for group 2 and 110% for group 3. The differences between the groups were statistically significant (*p* < 0.05) for groups 1 and 2 and groups 1 and 3, but no significant change for groups 2 and 3, as indicated in Table 4. Furthermore, the result of the interference screw method was compared with the CBPF’s third group, and the resulted mechanical properties of the two groups were significantly different (*p* < 0.05) (Table 4).

### Mode of failure

The mode of failure during the testing process was monitored visually. For most of the test samples in the CBPF technique (20 out of 30), the tendon slippage out of the tunnel was the mode of failure. In group 1 with low-density Sawbones samples, the fixation failed due to the tendon slippage at the interface with the core bone and tunnel wall. A different behaviour was observed for the test groups with higher density Sawbones (groups 2 and 3). In these two groups, the fixation of 50% of the test samples failed due to the tendon rupture. Also, the mode of failure in the interference screw method was due to the rupture of the connective tissue for all ten samples.

Detailed investigation of the results obtained from single cycle load to failure test shown that there is no significant difference (*p* > 0.05) between the mechanical stability results of the test samples associated with each failure mode. For instance, the means (and standard deviations) of the ultimate strengths of the tendon slippage (5 samples) and tendon rupture (5 samples) test samples of groups 2 and 3 were 313 N (±77.5 N) and 307 N (±86.7 N), and 366.6 N (±192.9 N) and 359.4 N (±103 N), respectively.

### Core bone integrity

**Fig. 11:**
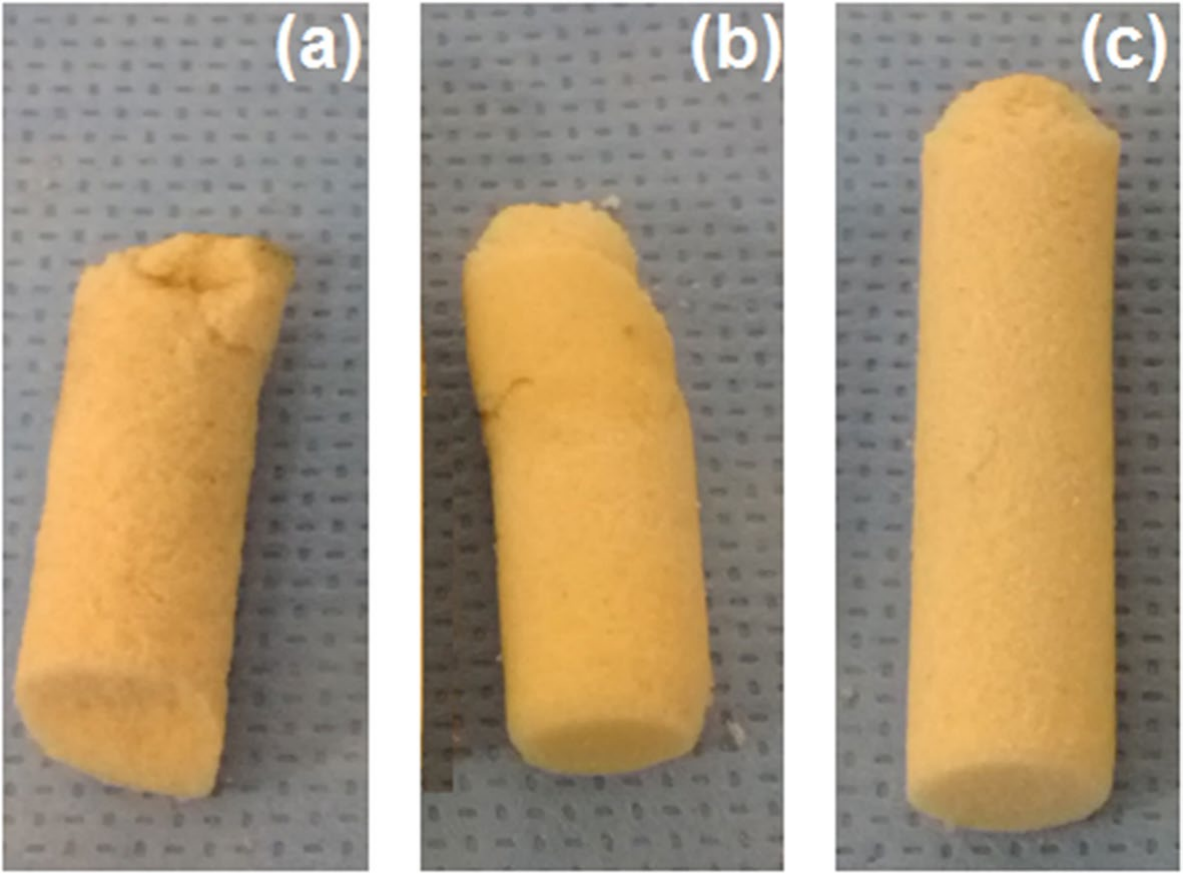
Typical samples of core bone after the experiments: (a) Low density (group 1) core bones experienced maximum damage and become shorter with many small fragments, (b): Middle density (group 2) core bones turned into relatively longer plugs with a lower number, (c): High density (group 3) core bones experienced minimum damage

Investigation of the overall integrity of the test samples after the pull-out tests revealed that the core bones experiencing damage during the insertion process. The maximum damage was observed for the low-density samples of group 1, in which the original core bone becomes shorter when the insertion force applies. This can be seen in Fig. 11. Local fracture at the contact zone between the insertion screw and the core bone head is the most obvious reason for this damage. Another reason can be fractures due to buckling during the insertion process. For group 2, the final core bone was relatively longer and occurred for the low number of specimens. Finally, for group 3, the change of the core bone shape during the insertion process was minimal.

## Discussion

### Biomechanical consideration

This biomechanical study investigates the effect of bone density on the primary stability of the ACL reconstruction using CBPF. While animal [16] and cadaver studies [28, 33, 34] have difficulty controlling bone density, using approved artificial as a substitute for fresh bone can solve the problem. The Sawbones blocks made it possible to produce controlled samples and examine those in groups with different densities. Our results show that bone density plays an important role in the primary stability of the CBPF technique, and the groups with 240 kg/m^3^ and 320 kg/m^3^ are appropriate for this method. As a result, the test samples made of low-density Sawbones (group 1) often experience larger amounts of graft slippages during the cyclic loading tests (Table 3). During the failure loading test, group 1 showed smaller stiffnesses when subjected to small loads, i.e., at the toe region or prior to the yielding point (Fig. 10 and Table 4). These observations suggest that the low-density bone plugs may not produce a stable fixation immediately after insertion. This is mainly because of the damages received by core bone (Fig. 11). During the insertion process, the core bone is fractured and split into several smaller parts, affecting the primary stability of the fixation. In fact, the experience of this study showed that it is practically challenging to insert a naked low-density core bone into a tunnel undamaged. The CBPF is thus not appropriate for low-density bones unless special surgical tools are available for plug insertion or the core bone armed and shielded, and this is a recommendation for future investigations. There is no such problem for higher density bones, and the bone plug has enough strength to remain intact during a conventional insertion process.

On the other hand, in the single-cycle load to failure tests performed in this study, all low-density (group 1) test samples failed due to the tendon slippage at the fixation site through the interface of the core bone and the tunnel wall; while 50% of the samples of higher density test groups (groups 2 and 3), failed because of the tendon rupture. Considering the fact that the ultimate strength of the bovine digital extensor tendons has been reported to be more than 1000 N [1], this observation suggests that the tendon fibres have been cut or damaged during the insertion process. Thus, the way core bone is inserted into the tunnel and its engagement with the tendon and tunnel wall is essential in the CBPF technique. It must be noted that the current study performed on artificial bone materials while in the use of fresh bone may change the condition as the fresh bone is more elastic and diluted with organic lubricants, e.g., fats, which may help for easier core bone insertion [16].

In addition, the mechanical properties of the interference screw were compared with group 3, which have the same Sawbones block densities (Table 4). As the statistical analysis indicates, the differences between the yield strength, ultimate strength, and stiffness for these two methods were significant (*p* < 0.05). On the other hand, the differences between the slippage values of the connective tissues in the CBPF technique (group 3) and interference screw were insignificant (*p* > 0.05). Therefore, we can say that the CBPF technique has had an appropriate performance in cyclic loading and daily post-rehabilitation activities and will not face any problem in this stage. Besides, it is noteworthy that the ultimate strengths of groups 2 and 3 were greater than the average forces applied to an intact ACL during normal walking (i.e., 169 N) or slope climbing (i.e., 67 N) [35]. Therefore, with more precaution during the early stage of rehabilitation, considering its numerous clinical advantages, the CBPF technique could be used as an alternative for ACL reconstruction.

The study performed by Bashti et al. [16] to simulate ACL fixation using fresh bone specimens and CBPF technique against absorbable interference screw fixation showed a close agreement between CBPF and interference screw primary fixation strengths. However, in that study, the intention was only to show the CBPF technique is a valid method, but their results cannot be used for human cases as in their tests performed using bovine bones which density is far stronger than human bone.

Considering the result obtained for the CBPF technique, a restricted rehabilitation immediately after the operation is suggested. It means care must be taken in the first few days after the surgery to avoid any overloading to the graft-bone construct. Nevertheless, despite the identical density of the test samples within each group, the ranges of the results for the stiffness, the yield strength, the ultimate strength of the fixation, and the graft slippage were wide. For instance, the ultimate strength of fixation varied between 205 and 702 N for the test samples of the high-density test group (Table 4). This observation suggests that there are other important factors, besides bone quality, that affect the primary fixation stability of the ACL grafts using CBPF. In particular, the size (thickness) of the graft in relationship with the diameter of the bone plug is thought to be a major factor affecting the stability of fixation and tendon integrity. To minimize this diversity and obtain repeatable results, the preparation technique of the graft and bone plug should be improved, and their size should be adjusted precisely. Furthermore, more sophisticated surgical tools should be developed for bone tunnelling and core bone removal from the cannulated drill bit. More specifically, an automated device may need to insert core bone into the tunnel. The insertion tool should cause minimum damage to the components and make the process feasible in the clinical environment.

### Clinical consideration

CBPF technique is an organic and implant-less fixation method applicable for ACL reconstruction. It was shown that bone-to-bone healing is faster compared to that techniques used interference screw for fixation. Also, other clinical advantages (e.g., higher primary stability, early functional rehabilitation, ease for revision surgery, no interferences in imaging, cost-effectiveness) for implant-less techniques were seen [17, 18].

Besides, Bonasia et al. showed that [2] bone-to-bone healing and soft tissue healing in a femoral or tibial tunnel in ACL reconstruction take almost 6 and 8–12 weeks after surgery, respectively. On the other hand, Weiler et al. [36] concluded that the biodegradable interference screw would macroscopically degrade after 24 weeks after surgery. Even after 52 weeks, the reconstruction’s tensile stress equals 47.3% of the native ACL. Also, it was mentioned that using an interference screw to fix the soft tissue may also alter the graft’s mechanical properties in the early remodeling stage at the insertion site.

In addition, Jagodzinski et al. [37] compared the press-fit technique with biodegradable interference screw for the healing process with respect to tunnel enlargement, and it was found that bone plug usage limits tunnel widening and likely enhances bone healing, and it is beneficial for ACL revision surgery.

However, before a clinical investigation, there are a need for possible in-vitro and in-vivo studies to elaborate on its biomechanical characteristics in more detail. This is particularly true for the primary stability of the graft fixation, which is thought to be significantly affected by the fixation method and bone quality. With a poor bone quality, i.e., low-density bone, a weak fixation is anticipated, not only during the insertion process but also at the immediate post-operation loadings stage. As a result, it might not be able to secure the graft with enough stability to initiate bone-graft integration. This is true when a naked core bone plug is used. Although, the researchers [25] showed that the condition could change if the core bone is sheathed.

On the other hand, a solid and rigid bone can cause damage to the soft tissue tendon graft and mechanical integrity of the structure, either during the insertion process or due to the post-operation loadings. Thus, while the fixation is strong, the graft itself is not capable of withstanding the loads applied to it after the surgery. With this regard, although bone plug with densities of 240 kg/m3 and 320 kg/m3 both can be taken into account for ACL reconstruction using the CBPF technique, higher density is preferred if the insertion process can be improved and the risk of damage on tendon fibres minimized. However, this can be a matter of concern that needs investigation before any clinical examination. The result of the current study can contribute to a deeper insight into the biomechanics of the ACL reconstruction using CBPF. It was our dream to provide guidelines for possible clinical examination, and therefore further investigation in this manner is encouraged.

### Improvement and future investigation

The main advantage of the CBPF is using the patient’s own bone for fixing the ACL graft, with no need for an external object as the fixation implant. This makes the donor protocols simpler and helps to minimize the risk of contamination. It is assumed to provide a faster healing process in comparison with the conventional implant-based methods. Unlike implanted methods that are subjected to external object side effects, blocking the contact zone between the bone and graft and damaging the bone during the fixation procedure [16–19], the CBPF technique provides minimum side effects. However, the healing process of the CBPF technique for the application of ACL reconstruction has not been studied yet. Further investigation is recommended on animal models to compare the secondary fixation stability of the grafts in the mid and long term when a CBPF ACL reconstruction technique is used.

Generally, in the insertion process of a core bone into a bony tunnel using a hammer, not the whole length of the core bone will be inserted into the tunnel. This is mainly because of the local fracture of the core bone at the contact zone with the hammer. It has been assumed that the inserted length of the core bone will affect the CBFC fixation strength and its healing time. Hence it is crucial to have comprehensive knowledge about the geometric effect of the engaged length of the core bone into the tunnel. Furthermore, the relationship between the tunnel size and the tendon’s diameter needs further investigation.

Although the outcome of the CBPF technique provides acceptable stability for young and middle-aged patients with good bone quality, to complete this achievement, the design of surgical tools should be developed to obtain a reproducible ACL reconstruction technique.

## Conclusions

This study uses the CBPF technique, an organic and implant-less method to fix soft tissue tendon into a bone tunnel for ACL reconstruction. The study investigates the bone density effect on the CBPF stability using a series of experimental investigations. Fresh animal tendon and artificial bone block were used in three different groups to fulfil the hypothesis of the study. The study concluded that the primary fixation stability of the grafts using the CBPF technique is greatly affected by bone density. Although this is not a clinical study, the result of this study can be used to improve the ACL reconstruction treatment method. The outcome of the current research suggests that CBPF can be an alternative ACL reconstruction technique for patients with good bone quality.

## Data Availability

The datasets used and/or analysed during the current study are available from the corresponding author on reasonable request.
